# Induced abortion on demand and birth rate in Sami-speaking municipalities and a control group in Finnmark, Norway

**DOI:** 10.3402/ijch.v72i0.20357

**Published:** 2013-05-14

**Authors:** Jan Norum, Tove E. Svee, Anca Heyd, Carsten Nieder

**Affiliations:** 1 Institute of Clinical Medicine, Faculty of Health Sciences, University of Tromsø, Tromsø, Norway; 2 Northern Norway Regional Health Authority trust, Bodø, Norway; 3 Department of Oncology, University Hospital of North Norway, Tromsø, Norway; 4 Division of Oncology and Palliative Care, Department of Internal Medicine, Nordland Hospital, Bodø, Norway

**Keywords:** Sami, abortion, birth, Norway

## Abstract

**Objectives:**

The objective of this study was to analyze the birth and induced abortion on demand (IAD) rate among women in Sami-speaking communities and a control group in Finnmark County, Norway.

**Methods:**

The 6 northern municipalities included in the administration area of the Sami language law (study group) were matched with a control group of 9 municipalities. Population data (numbers, sex and age) were accessed from Statistics Norway. Data on birth rate and IAD during the time period 1999–2009 were derived from the Medical Birth Registry (MBR) of Norway. Data on number of women in fertile age (15–44 years) were obtained from Statistics Norway. Between 2001 and 2008, this age group was reduced by 12% (Sami) and 23% (controls), respectively.

**Results:**

Finnmark County has a high IAD rate and 1 in 4 pregnancies (spontaneous abortions excluded) ended in IAD in the study and control groups. The total fertility rate per woman was 1.94 and 1.87 births, respectively. There was no difference between groups with regard to the IAD/birth ratio (P=0.94) or general fertility rate GFR (P=0.82).

**Conclusions:**

Women in the Sami-majority area and a control group in Finnmark County experienced a similar frequency of IAD and fertility rate.

The Sami have their own language and culture. Traditionally the lifestyle diverges from that of the rest of the Norwegian population, but in relation to occupational expansion, traditions are changing. Until recently, research to understand health issues specifically for the Sami peoples has been lacking. No systematic registration of ethnicity that can be used for research purposes has existed since the 1970 Census.

The Sami live in the northern regions of Norway, Sweden, Finland and Russia's Kola Peninsula. The Norwegian government has ratified the Sami as the indigenous people in Norway (1). The exact number of Sami is not known. The Sami population has been estimated to be approximately 75,000–100,000, but estimates vary depending on the criteria used, such as genetic heritage, mother tongue and sense of belonging to the Sami. According to the definitions employed by the Sami Parliament, a Sami is a person who speaks Sami, or who has one of the parents, grandfathers/grandmothers or great grandfathers/grandmothers who spoke a Sami language.

In Norway, all inhabitants have equal rights to health care (2). Norwegian health care authorities have been concerned about offering the Sami minority and Norwegians in general the same high quality health care service (2–4). The concern on the Sami health care has been based on prior research revealing that Sami people are not satisfied with the health care service offered (5).

Cultural and linguistic differences may affect Sami's access to health care (6). The challenges have often been summarized as threshold, counter, queue and cultural challenges. These hindrances may influence requests and access to induced abortion on demand (IAD). Furthermore, Sami have been documented as having a significantly lower incidence of breast cancer (7–10). Life-time risk of breast cancer is correlated with genetic heritage and hormonal factors. Age at menarche, age at first pregnancy, number of pregnancies, and age at menopause, body mass index, hormonal replacement therapy, alcohol abuse and irradiation are all known risk factors (11–14). Based on these facts, we aimed to clarify the incidence of IAD and birth rate in the Sami-speaking municipalities and a selected control group in Finnmark County as these factors could potentially affect the risk of breast cancer.

## Material and methods

**Fig. 1:**
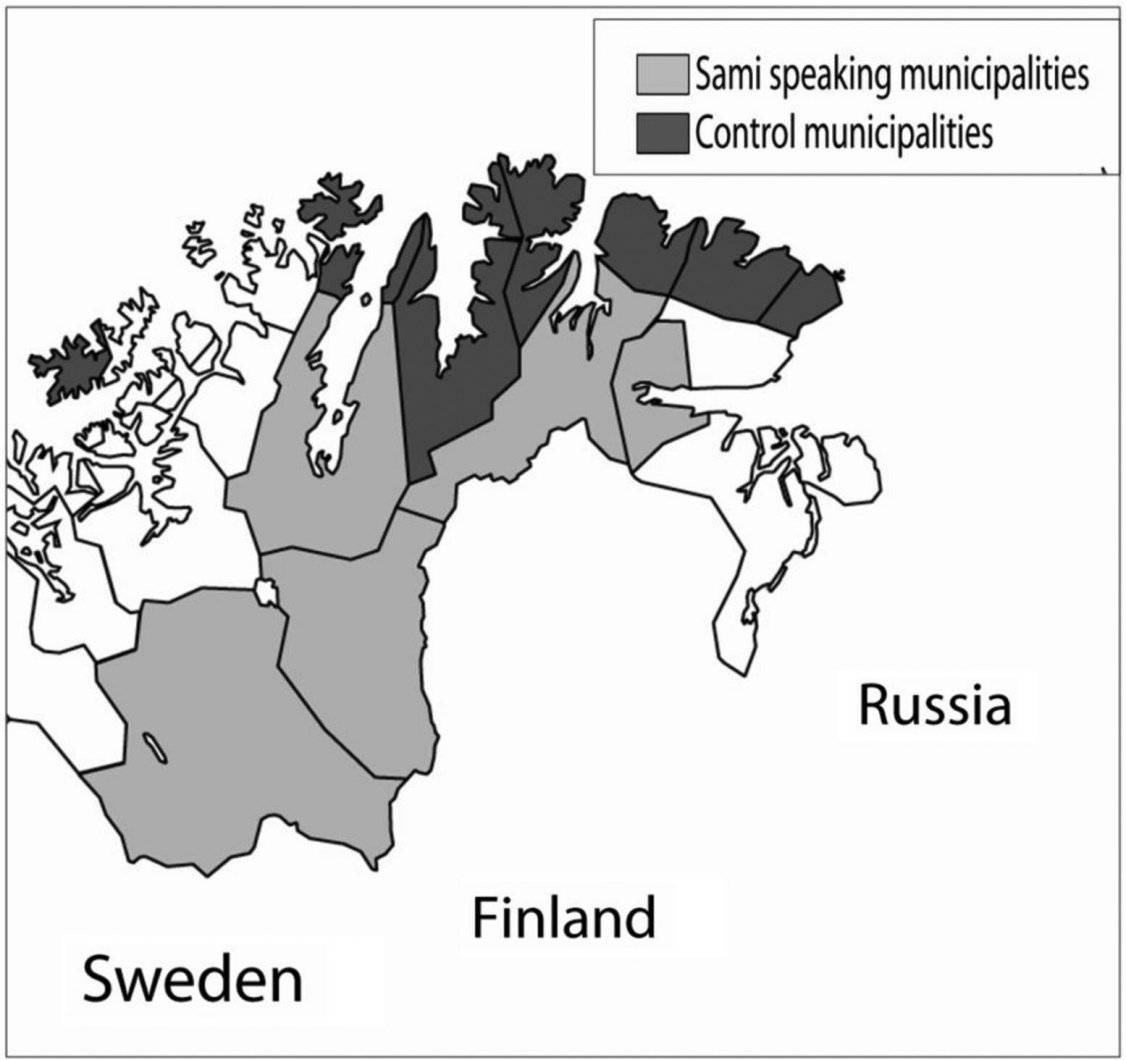
The figure shows Finnmark County with the municipalities in the Sami and control group, respectively.

Whereas 40 Norwegian municipalities have Sami residents, the percentage of Sami-speakers has only been analyzed in the municipalities of Finnmark County. In October 2000, a Gallup poll asked people if they could speak Sami (3). Five municipalities in Finnmark have been included in the administration area of the Sami language law. They were selected as the *study group* and the percentage of Sami-speakers was 71% in this group. The coastal municipalities of Finnmark have generally few Sami people and 8 of them were chosen as the *control group*. In the Gallup poll, 6% reported being Sami-speakers in this group. Details on location and population of the Sami and the control group are shown in Fig. 1 and Table I. The municipalities of the Sami group were Deatnu/Tana, Unjárga/ Nesseby, Porsanger/Porsángu/Porsanki, Kárásjohka/Karasjok and Guovdageaidnu/Kautokeino. The ones included in the control group (non-Sami group) were Lebesby, Gamvik, Måsøy, Båtsfjord, Berlevåg, Nordkapp, Hasvik, and Vardø. Characteristics of the municipalities in terms of population, age groups and gender were derived from Statistics Norway (www.ssb.no) and the 2001 and 2008 figures were employed. When calculating the general fertility rate (GFR), the mean figure 2001–2008 was employed. The number of women aged 15–44 years in the Sami and control group dropped from 2001 to 2008 by 12 and 23%, respectively. Details are shown in Table I. The change was mainly due to migration, but exact figures were not available.

**Table I:** The table shows number of women aged 15–44 years in the Sami-speaking municipalities and the control group. The numbers of inhabitants are according to the 2001 and 2008 figures from Statistics Norway (www.ssb.no)

Sami group	2001	2008	Mean	Control group	2001	2008	Mean
Tana	590	544	567	Vardø	473	346	410
Nesseby	161	145	153	Hasvik	223	157	190
Kautokeino	665	589	627	Nordkapp	639	566	603
Karasjok	591	522	557	Båtsfjord	528	379	454
Porsanger	854	723	789	Lebesby	269	210	240
				Berlevåg	235	157	196
				Måsøy	238	214	226
				Gamvik	239	169	204
Total	2,861	2,523	2,693	Total	2,844	2,198	2,523

### Statistical analysis and authorization

No individual patient data were analyzed. Anonymous and aggregated data (annual births and IADs) for each municipality were imported from the Medical Birth Registry (MBR) of Norway to the study database. Data on spontaneous abortions were not available and consequently not included in the analysis. The Microsoft Excel 2007 version was employed for the final database, calculations and statistical analysis. Descriptive statistics and the *t*-test were used for the comparison between groups. Statistical significance was set to 5%. The *t*-test was carried out 2-sided. The study was performed as a “quality of care analysis” as we aimed to clarify the 2 groups’ access to health care services in terms of IAD and delivery units. We had, as mentioned, no access to any individual patient data and consequently no approval from the Regional Committees for Medical and Health Research Ethics (REK) was necessary. Similarly, no approval from the Norwegian Social Science Data Services (NSD) was requested.

## Results

**Fig. 2:**
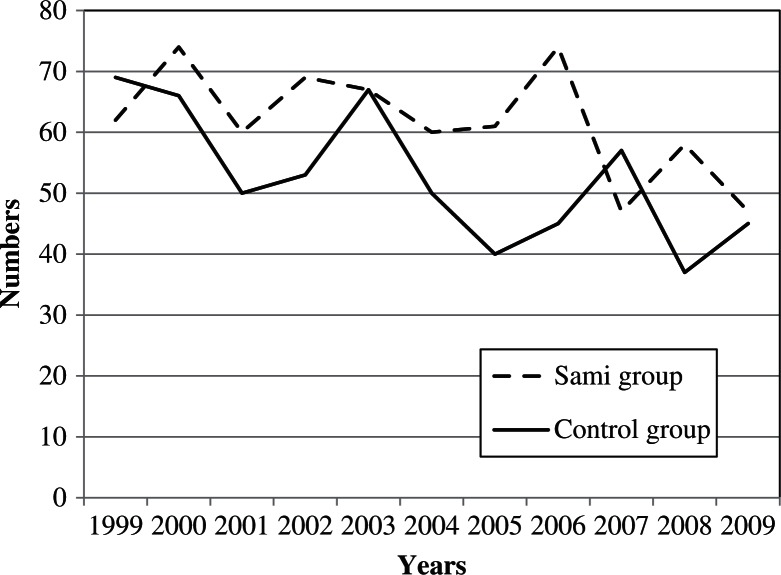
The figure shows the annual number of induced abortions on demand in the Sami and the control group.

**Fig. 3:**
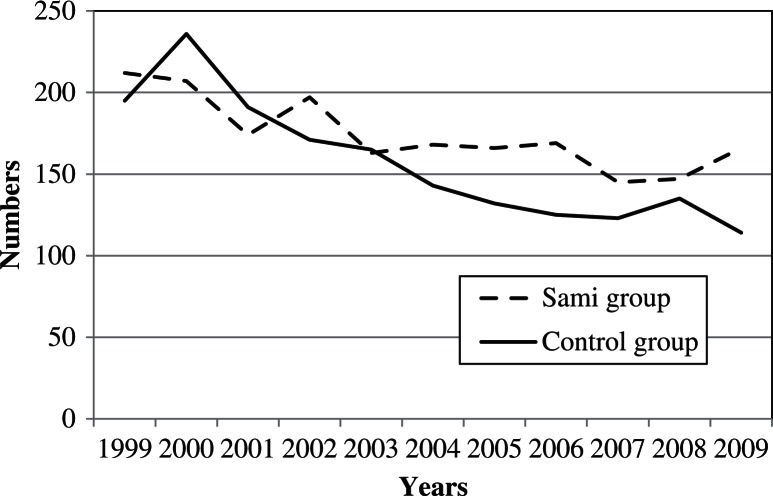
The figure illustrates the number of births annually in the Sami and the control group in the time period 1999–2009.

**Fig. 4:**
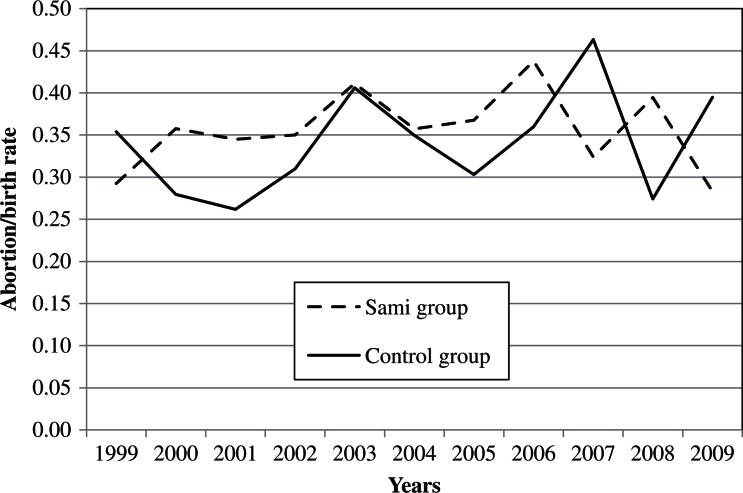
The figure shows the induced abortion on demand/births in the Sami and the control group during the time period 1999–2009. There was no significant difference (P=0.57).

During the 11-year study period (1999–2009), a total of 679 and 579 abortions were detected in the study and the control group, respectively. The corresponding number of births was 1,914 and 1,730 births, respectively. Whereas the absolute number of births had fallen during the study period (due to fewer women in the fertile age), the IAD/birth ratio was stable (Figs. 2–4). Whereas every fourth pregnancy (spontaneous abortions excluded) ended in an IAD, there was no significant difference (P=0.94) between groups. However, the ratio was higher in some municipalities. In Hasvik, Karasjok and Vardø, every third pregnancy (spontaneous abortions excluded) ended in IAD. Details are shown in Table II.

**Table II:** The table shows the number of births, abortions, women aged 15–44 years and the general fertility rate (GFR) in the Sami and control group during the timer period 1999–2009

	Abortions	Births	Abortion/Birth	Females aged 15–44 years	GFR*
Tana	85	378	0,22	567	60,6
Nesseby	20	84	0,24	153	49,9
Karasjok	215	388	0,55	557	63,3
Kautokeino	161	495	0,33	627	71,8
Porsanger	198	569	0,35	789	65,6
Total Sami	679	1,914	0,35	2,693	64,6
Vardø	139	296	0,47	410	65,6
Hasvik	60	123	0,49	190	58,9
Nordkapp	132	423	0,31	603	63,8
Båtsfjord	93	325	0,29	454	65,1
Berlevåg	35	146	0,24	196	67,7
Lebesby	46	167	0,28	240	63,3
Gamvik	30	119	0,25	204	53,0
Måsøy	44	131	0,34	226	52,7
Total control group	579	1,730	0,33	2,523	62,3

* GFR=general fertility rate=births/year/number of women aged 15–44 years*1,000.

The GFR [(births/year/women aged 15–44 years)*1,000] was similar in both groups. The values were 64.6 and 62.3 (P=0.82), respectively. Total fertility rate per woman was calculated as 1.94 and 1.87 births, respectively.

### Sensitivity analysis

A total of 29% of the population in the Sami group were ethnic Norwegians. In a model-based sensitivity analysis, we assumed this subgroup had the same birth and IAD rate as controls (96% ethnic Norwegians). Similarly, we calculated the Sami (6%) in the control group having the same experience as the Sami group. These corrections did not alter the finding of no difference between groups (P=0.412). Furthermore, no between-group difference was observed when we increased the share of ethnic Norwegians in the Sami group (39%) (P=0.411) and raised the percentage of Sami in the control group (16%) (P=0.415).

## Discussion

In this study, we have disclosed a high frequency of IAD in Finnmark. Furthermore, we documented that the Sami-speaking municipalities had a similar IAD and birth rate as the control group. Consequently, Sami municipalities have the same fertility rate as the other municipalities in the region.

The study was based on the fertile female population of 2 groups of municipalities in Finnmark County in Norway. Data were extracted from MBR of Norway and Statistics Norway. These registries are known for their high quality. All Norwegian hospitals and medical doctors by law have to report any case of IAD or birth to the national MBR.

In Norway, data on minorities such as the Sami people are not available, as we are not allowed to register people based on ethnicity. In this study, women in Finnmark living in the administration area of the Sami language law were selected as a surrogate for the Sami people. Whereas there are, without any doubt, a high percentage of Sami people in these municipalities, the exact percentage is unknown. Similarly, the percentage of Sami people among the control group consisting of coastal municipalities is low. However, the exact percentage is unknown, but the Gallup poll did document a significant difference in the share of Sami-speakers between groups (71% vs. 6%).

The unlinked analysis involved determining the number of incidents (IAD and birth) in each subgroup from the MBR of Norway (the numerator) and the number of people in each subgroup from census data (the denominator). A cross-sectional unlinked study is susceptible to numerator–denominator bias because of differences in the way the place of living is recorded in the MBR of Norway and census data from Statistics Norway. Whereas data on municipality levels are recorded as of January 1st each year, the MBR registers the mother's place of living at the time of delivery. Consequently, migration differences between groups may be a potential cause of bias.

The 2 groups differ in geographical setting. Whereas the Sami group is located inland, the control group is located in the coastal areas. However, both groups are rural.

We had no access to the exact number of women aged 15–44 years living in the 2 municipality-groups each year. Consequently, we did not calculate annual GFR figures. However, we believe the population data for 2001 and 2008 together give a good overview of the total time period.

We employed anonymous and aggregated data for each municipality imported from the MBR of Norway. Consequently, we could not make sub-analyses on women's age at pregnancy, IAD and delivery. Such sub-analyses would have been of interest as young age at first pregnancy is associated with a reduced risk of breast cancer.

IAD has been legalized in most Western countries during the last decades. Since 1979, women in Norway have had the right to have an IAD performed. Despite the introduction of new contraceptives and several campaigns in the junior high school and high school to educate young Norwegians on the use of contraceptives, the annual abortion figures have been constant. This was also observed in our study. At present, every fifth known pregnancy (spontaneous abortions excluded) in Norway is terminated by an induced abortion (15). Our study has documented the ratio in Finnmark (1:4) being above the national figures. The corresponding figures in Denmark, Sweden and England have been reported 1:6, 1:4, and 1:5, respectively (16). A similar figure (22 per 100 pregnancies) has been reported from the United States (17, 18). If a national strategy to reduce abortion is to be launched, Finnmark County could (based on our findings) be a suitable pilot site. Societies with an aging population may consider reducing abortion on demand as a social response to increase the proportion of children (19).

Most western countries have experienced a drop in fertility rate following the introduction of IAD 19. In Europe, in 2005, only Turkey, Iceland and Albania had total fertility rates above 2.0 children/women and only Finland, United Kingdom, Ireland, France, Denmark and Norway reported figures above 1.8 (www.epp.eurostat.ec.europa.eu). The average total fertility rate in the European Union (EU-27) was calculated at 1.59 children per woman in 2009. The 2 groups of municipalities in Finnmark have rates of 1.94 and 1.87. Without immigration or a significant drop in mortality, this figure will cause a future drop in the population of Finnmark County (2.1 is the reproduction rate necessary to maintain the population with today's mortality figures).

In this study, we observed that more women in the fertile age (15–44 years) migrated out of the municipalities in the control group (23% vs. 12%). This trend has also been documented by Statistics Norway (20). Traditionally, Sami municipalities have been more stable with few people moving out. This may be due to the fact that several important institutions offering jobs to the Sami people have been located to the Sami region. Examples include the Sami Parliament in Karasjok and the Sami College in Kautokeino.

Our original intent to study IAD and reduced fertility as a potential explanation for a lower breast cancer risk among Sami cannot be pursued as the 2 groups do not differ in terms of their IAD/birth ratios or fertility rates.
